# Tofacitinib and Baricitinib in Type 2 Diabetic Patients with Rheumatoid Arthritis

**DOI:** 10.3390/medicina60030360

**Published:** 2024-02-21

**Authors:** Cristina Martinez-Molina, Cesar Diaz-Torne, Hye S. Park, Anna Feliu, Silvia Vidal, Hèctor Corominas

**Affiliations:** 1 Department of Pharmacy, Hospital de la Santa Creu i Sant Pau, 08041 Barcelona, Spain; 2 Department of Medicine, Universitat Autònoma de Barcelona (UAB), 08193 Barcelona, Spain; 3 Department of Rheumatology and Systemic Autoimmune Diseases, Hospital de la Santa Creu i Sant Pau, 08041 Barcelona, Spain; 4 Group of Immunology-Inflammatory Diseases, Sant Pau Biomedical Research Institute (IIB Sant Pau), 08041 Barcelona, Spain

**Keywords:** Janus kinase inhibitor, tofacitinib, baricitinib, rheumatoid arthritis, type 2 diabetes mellitus

## Abstract

*Background and Objectives*: Recently, a randomized controlled trial suggested a potential benefit of baricitinib in patients with diabetes mellitus, preserving β-cell function. However, the clinical evidence currently available is limited. We aimed to assess the potential impact of tofacitinib and baricitinib on type 2 diabetes mellitus (T2DM) patients with rheumatoid arthritis. *Materials and Methods*: The candidates for this observational, retrospective, single-center study were selected from a cohort of 120 rheumatoid arthritis patients treated with tofacitinib or baricitinib between September 2017 and September 2023. The eligibility criteria included patients with T2DM who were receiving oral antidiabetic drugs (OADs). The primary outcome was the glycosylated hemoglobin (HbA1c) value after 6 months of a JAK inhibitor treatment. Secondary outcomes included body mass index (BMI) and rheumatoid arthritis disease activity. Differences were evaluated using Fisher’s exact test, as well as the Mann–Whitney test or the Wilcoxon test. *Results*: Thirteen patients were included; 46.2% (6/13) underwent treatment with tofacitinib, while 53.8% (7/13) were treated with baricitinib. At 6 months, baricitinib treatment resulted in a reduction in HbA1c (*p* = 0.035), with 57.1% (4/7) of patients achieving values <7%, and 28.6% (2/7) of patients requiring a reduction in OAD dosage. Concerning BMI, an increase (*p* = 0.022) was observed at 6 months following baricitinib administration. All the patients treated with either tofacitinib or baricitinib achieved remission or low disease activity, without requiring statistically significant changes in concomitant rheumatoid arthritis treatment. *Conclusions*: In T2DM patients with rheumatoid arthritis, baricitinib can improve insulin sensitivity and glucose uptake, enabling the optimization of T2DM management.

## 1. Introduction

Janus Kinase (JAK) inhibitors are small molecules designed to modulate the JAK and Signal Transducer and Activator of Transcription (STAT) signaling pathways. These drugs exhibit specific profiles of JAK inhibition [1] and are used to treat moderate to severe active rheumatoid arthritis [2] and other immune-mediated inflammatory diseases. The first generation of JAK inhibitors, characterized by a low selectivity profile, includes tofacitinib and baricitinib. Tofacitinib primarily acts on JAK1 and JAK3 and to a lesser extent on JAK2 [1]. Baricitinib predominantly inhibits JAK1 and JAK2 [1].

The activation of the JAK-STAT pathway can occur aberrantly through interference with the insulin signaling pathway [3,4,5]. Insulin signal reception can lead to the phosphorylation of JAK2 through the tyrosine kinase coupled with the receptor of this hormone, disrupting glucose uptake signaling [4,5]. The activation of the JAK2-STAT1 pathway is associated with the impairment of pancreatic β-cells, promoting the development of diabetes mellitus [3,5]. Recently, a randomized controlled trial suggested that baricitinib could preserve β-cell function in type 1 diabetes mellitus patients [6]. JAK2 is also involved in leptin signaling, the recognition of which activates the JAK2-STAT3 pathway, contributing to appetite suppression and increased energy expenditure [5,7]. 

According to the recommendations of the European Medicines Agency (EMA), special caution should be taken when prescribing a JAK inhibitor to a rheumatoid arthritis patient with diabetes mellitus due to an increased risk of developing infections [8,9]. This fact elucidates the limited number of patients with these characteristics undergoing treatment with tofacitinib or baricitinib in real-world clinical practice.

Through the present study, we aim to assess the potential impact of tofacitinib and baricitinib, two JAK inhibitors with different JAK2 inhibition profiles, on type 2 diabetic patients with rheumatoid arthritis.

## 2. Materials and Methods

This was an observational, retrospective, single-center study, conducted at a community-based university hospital, that involved real-world patients (aged ≥ 18 years) who fulfilled the 2010 American College of Rheumatology (ACR)—European League Against Rheumatism (EULAR) classification criteria for rheumatoid arthritis [10]. Type 2 diabetic patients receiving oral antidiabetic drugs (OADs) were eligible for inclusion in this study. All included patients were individually informed about the study protocol and were given the option to decline participation in data extraction. All patients were treated with either tofacitinib or baricitinib between September 2017 and September 2023. 

The primary outcome was the glycosylated hemoglobin (HbA1c) value after 6 months of treatment with either tofacitinib or baricitinib. Secondary outcomes included body mass index (BMI) and rheumatoid arthritis disease activity. 

At baseline, demographic and clinical patient characteristics were separately detailed according to JAK inhibitor type. For each JAK inhibitor, variables were described prior to the JAK inhibitor treatment (0 month) and at 6 months of treatment. Categorical variables were expressed as absolute number (*n*) and percentage (%). Ordinal and quantitative variables were presented using the median and interquartile range (IQR: [P25–P75]). The differences were evaluated using Fisher’s exact test (for independent categorical variables), as well as the Mann–Whitney test or the Wilcoxon test (for independent or related ordinal and quantitative variables). The statistical analyses were performed utilizing Stata software version 12. A *p*-value of <0.05 was considered statistically significant.

For assessing rheumatoid arthritis disease activity, the following scales were considered: Disease Activity Score 28 (DAS28), using erythrocyte sedimentation rate (DAS28-ESR); DAS28, using C-reactive protein (DAS28-CRP); Clinical Disease Activity Index (CDAI); and Simplified Disease Activity Index (SDAI). Rheumatoid arthritis disease activity was classified according to the updated recommendations provided by the American College of Rheumatology [11] into remission, low disease activity (LDA), moderate disease activity (MDA), and high disease activity (HDA). 

This study received approval from the ethics committee of a hospital (IIBSP-JAG-2023-168). This study, which involved human participants, adhered to the principles of the 1964 Helsinki Declaration and its later amendments or comparable ethical standards.

## 3. Results

From a cohort of 120 rheumatoid arthritis patients treated with JAK inhibitors between September 2017 and September 2023, 10.8% (13/120) were selected due to their concurrent treatment with OADs for type 2 diabetes mellitus. With regard to the selected patients, 46.2% (6/13) were undergoing treatment with tofacitinib, while 53.8% (7/13) were undergoing treatment with baricitinib. Their demographic and clinical characteristics are summarized in Table 1. Upon the JAK inhibitor treatment’s initiation, the tofacitinib and baricitinib treatment groups showed comparable ages, sex distribution, rheumatoid arthritis disease activity scores, prior biologic Disease Modifying Anti-Rheumatic Drug (bDMARD) use, concomitant rheumatoid arthritis treatment, HbA1c values, type 2 diabetes mellitus treatment, and BMI values.

**Figure 1:**
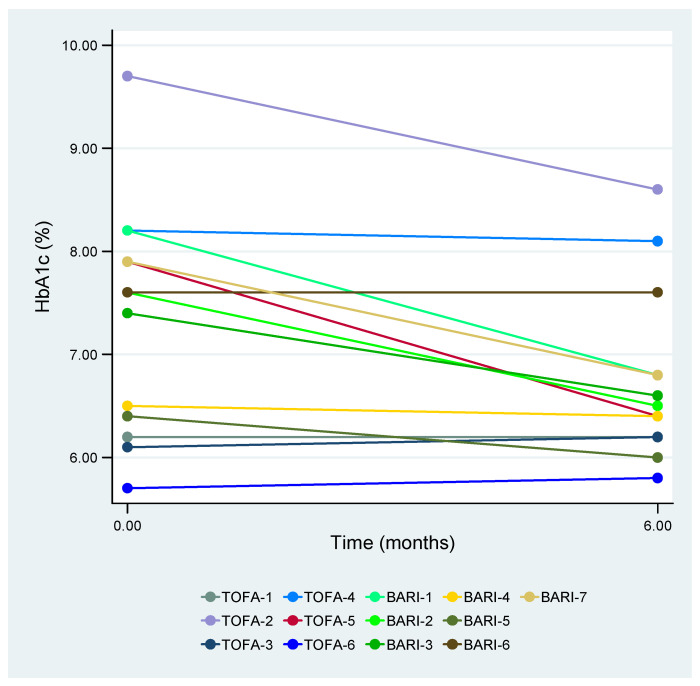
HbA1c values after 6 months of treatment with tofacitinib or baricitinib. HbA1c—glycosylated hemoglobin, TOFA—tofacitinib, and BARI—baricitinib.

The main findings following 6 months of treatment with tofacitinib and baricitinib are presented in Table 2 and Table 3, respectively. In terms of HbA1c values, tofacitinib showed no significant differences (*p* = 0.416), whereas baricitinib demonstrated significant decreases (*p* = 0.035). At 6 months, 57.1% (4/7) of the patients treated with baricitinib achieved HbA1c values <7%, in contrast to 16.7% (1/6) in the tofacitinib group (Figure 1). Treatment monitoring revealed, on the one hand, no statistically significant differences in terms of variations in concomitant GC doses in either the tofacitinib or baricitinib groups. On the other hand, a reduction in OAD dosage was observed for 28.6% (2/7) of the patients treated with baricitinib, while no changes were noted for the remaining 71.4% (5/7). With respect to BMI, the patients treated with baricitinib exhibited a significant increase from 28.7 (26.8–34.0) kg/m^2^ to 29.9 (27.2–34.0) kg/m^2^ at 6 months (*p* = 0.022). No significant differences in BMI were observed in the tofacitinib treatment group. Concerning rheumatoid arthritis disease activity, all the patients responded to the treatment, achieving either remission or at least LDA within 6 months following tofacitinib or baricitinib, without requiring statistically significant changes in the concomitant rheumatoid arthritis treatment.

## 4. Discussion

This study assessed the potential impact of tofacitinib and baricitinib, two JAK inhibitors with different JAK2 inhibition profiles, on type 2 diabetic patients with rheumatoid arthritis. Based on the published literature, there is limited research addressing this topic from the perspective of the clinical practice. Cases of hypoglycemia have been reported among diabetic patients with rheumatoid arthritis who have been treated with JAK inhibitors [12]. Recently, a phase 2 randomized controlled trial suggested that treatment with baricitinib preserves β-cell function in type 1 diabetes mellitus patients [6]. 

We present findings from seven cases of tofacitinib treatment and six cases of baricitinib treatment administered to type 2 diabetic patients within a cohort of 120 rheumatoid arthritis patients who were treated with JAK inhibitors at our community-based university hospital between September 2017 and September 2023. Following the EMA recommendations [8,9], a limited number of type 2 diabetic patients with rheumatoid arthritis were treated with tofacitinib or baricitinib. 

Concerning HbA1c, after 6 months of treatment, values significantly decreased in the baricitinib group (*p* = 0.035) but not in the tofacitinib group (*p* = 0.416). Without variations in concomitant GC doses, 57.1% of the patients treated with baricitinib achieved HbA1c values <7%. Additionally, a reduction in OAD dosage was necessary for 28.6% of patients treated with baricitinib. Collota D et al. [13], in the wake of analyzing the response to baricitinib treatment in a murine model administered a high-fat and high-carbohydrate diet, concluded that JAK2 inhibition can restore insulin signaling. In a murine model in which type 2 diabetes mellitus was induced, Bako HY et al. [14] observed that tofacitinib, in combination with acetylsalicylic acid, mitigated insulin resistance and, consequently, hyperglycemia. The interferon (IFN)-γ/pSTAT1 pathway, related to the impairment of pancreatic β-cells in type 2 diabetes mellitus [3,5] and involved in the pathogenesis of rheumatoid arthritis [1], is primarily mediated by JAK2 [1]. Baricitinib induces greater inhibition of IFN-γ/pSTAT1 compared to tofacitinib [1]. Thus, the potency of JAK2 inhibition could explain the magnitude of the observed differences.

With respect to BMI, weight gain was evidenced after 6 months of baricitinib treatment (*p* = 0.022) but not treatment with tofacitinib (*p* = 0.159). The inhibition of JAK2, due to its involvement in the JAK2-STAT3 pathway, could be a reason for the weight gain experienced by the patients treated with baricitinib. Bates SH et al. [15] emphasized the importance of signaling through STAT3 in the regulation of leptin, with its suppression being a cause of obesity.

Regarding rheumatoid arthritis disease activity, without requiring statistically significant changes in the concomitant rheumatoid arthritis treatment, all patients responded to the JAK inhibitor treatment, achieving either remission or at least LDA within the 6 months following tofacitinib or baricitinib. These findings corroborate that tofacitinib and baricitinib exhibit similar treatment effectiveness [1], with significant improvements in rheumatoid arthritis disease activities at 6 months of treatment.

As a limitation of the present study, the small number of patients should be taken into account when extrapolating the results obtained to the broader population, despite their concordance with previously reported evidence. 

The main strength of our study lies in being the first that, in real-world clinical practice, aimed to elucidate the transcendence of JAK2 inhibition in type 2 diabetic patients with rheumatoid arthritis.

## 5. Conclusions

In summary, our study suggests that for type 2 diabetic patients with rheumatoid arthritis, baricitinib can offer several advantages over tofacitinib. These advantages extend to improvements in insulin sensitivity and glucose uptake, enabling the optimization of type 2 diabetes mellitus management, with reductions in OAD requirements in some of those patients treated with baricitinib. 

## Figures and Tables

**Table 1:** Demographic and clinical characteristics upon initiating the JAK inhibitor treatment.

Parameters	TOFA (*n* = 6; 46.2%)	BARI (*n* = 7; 53.8%)	*p*-Value
Age (years), median [IQR]	74 [62–80]	71 [58–73]	0.566
Sex (female), *n* (%)	5 (83.3)	4 (57.1)	0.559
RA disease activity score			
DAS28-ESR, median [IQR]	4.8 [3.6–5.1]	5.1 [4.3–5.1]	0.721
DAS28-CRP, median [IQR]	4.6 [4.2–4.6]	4.2 [3.6–4.5]	0.391
CDAI, median [IQR]	21.5 [15.0–23.0]	16.0 [15.0–22.0]	0.614
SDAI, median [IQR]	21.2 [16.2–26.2]	16.4 [14.1–25.8]	0.668
Previous bDMARDs			
Adalimumab, *n* (%)	2 (33.3)	1 (14.29)	0.559
Certolizumab, *n* (%)	3 (50.0)	2 (28.6)	0.573
Etanercept, *n* (%)	4 (66.7)	3 (42.9)	0.592
Golimumab, *n* (%)	1 (16.7)	0 (0.0)	0.462
Infliximab, *n* (%)	6 (100)	7 (100)	-
Tocilizumab, *n* (%)	4 (66.7)	3 (42.9)	0.592
Sarilumab, *n* (%)	0 (0.0)	2 (28.6)	0.453
Abatacept, *n* (%)	2 (33.3)	1 (14.29)	0.559
Rituximab, *n* (%)	0 (0.0)	1 (14.29)	1.000
RA treatment			
GC, *n* (%)	4 (66.7)	4 (57.1)	1.000
PDN dose equivalent (mg/day), median [IQR]	5.0 [0.0–5.0]	5.0 [0.0–5.0]	0.735
csDMARD			
MTX, *n* (%)	2 (33.3)	2 (28.6)	1.000
MTX dose (mg/week), median [IQR]	0.0 [0.0–5.0]	0.0 [0.0–5.0]	0.859
Other csDMARD, *n* (%)	0 (0.0)	0 (0.0)	-
T2DM monitoring			
HbA1c (%), median [IQR]	7.1 [6.1–8.2]	7.6 [6.5–7.9]	0.774
T2DM treatment			
OAD use, *n* (%)	5 (83.3)	5 (71.4)	1.000
OAD use + insulin use, *n* (%)	1 (16.7)	2 (28.6)	1.000
BMI (kg/m^2^), median [IQR]	26.9 [22.6–32.2]	28.7 [26.8–34.0]	0.520

Differences were evaluated utilizing Fisher’s exact test or the Mann–Whitney test. *p*-value < 0.05. TOFA—tofacitinib, BARI—baricitinib, IQR—interquartile range [P25–P75], RA—rheumatoid arthritis, DAS28-ESR—Disease Activity Score 28-joint count using Erythrocyte Sedimentation Rate, DAS28-CRP—Disease Activity Score 28-joint count using C-Reactive Protein, CDAI—Clinical Disease Activity Index, SDAI—Simplified Disease Activity Index, bDMARD—biologic disease-modifying anti-rheumatic drug, GC—glucocorticoid, PDN—prednisone, csDMARD—conventional synthetic disease-modifying antirheumatic drug, MTX—methotrexate, T2DM—type 2 diabetes mellitus, HbA1c—glycosylated hemoglobin, OAD—oral antidiabetic drug, and BMI—body mass index.

**Table 2:** Main findings following 6 months of treatment with tofacitinib.

Parameters	TOFA-1	TOFA-2	TOFA-3	TOFA-4	TOFA-5	TOFA-6	*p*-Value
Sex	F	F	F	F	M	F	
Age (years)	80	53	62	71	77	82	
HbA1c (%)							
Prior to TOFA	6.2	9.7	6.1	8.2	7.9	5.7	0.416
At 6 months	6.2	8.6	6.2	8.1	6.4	5.8	
AD dosage	NA	NA	NA	NA	NA	NA	
Prior to TOFA	M850/24	M1/12 + S50/12	M850/12	M850/24 + E10/24 + IAI	M850/12 + S50/12	M850/12	
At 6 months	M850/24	M1/12 + S50/12	M850/12	M850/24 + E10/24 + IAI	M850/12 + S50/12	M850/12	
BMI (kg/m^2^)							
Prior to TOFA	24.0	29.7	32.2	34.0	20.7	25.6	0.159
At 6 months	24.0	30.4	32.2	34.0	21.9	25.6	
RA disease activity							
Prior to TOFA	MDA	MDA	MDA	MDA	HDA	HDA	-
DAS28-ESR	3.61	4.52	5.05	3.55	5.10	5.51	
DAS28-CRP	4.17	4.60	4.53	3.03	4.62	4.62	
CDAI	22.00	21.00	15.00	11.00	33.00	23.00	
SDAI	16.20	25.10	17.30	12.10	38.60	26.20	
At 6 months	Rem	LDA	Rem	Rem	LDA	LDA	
DAS28-ESR	1.19	3.15	2.49	1.81	3.17	2.90	
DAS28-CRP	1.75	2.75	1.17	1.52	2.28	2.16	
CDAI	2.00	10.00	1.00	2.00	8.00	8.00	
SDAI	3.10	10.20	0.80	3.30	10.60	10.50	
RA treatment							
GC (mg/day)							
Prior to TOFA	0.0	0.0	5.0	5.0	5.0	5.0	0.157
At 6 months	0.0	0.0	2.5	2.5	5.0	5.0	
MTX (mg/week)							
Prior to TOFA	5.0	0.0	5.0	0.0	0.0	0.0	-
At 6 months	5.0	0.0	5.0	0.0	0.0	0.0	

Differences were evaluated utilizing the Wilcoxon test. *p*-value < 0.05. TOFA—tofacitinib, F—female, M—male, HbA1c—glycosylated hemoglobin, AD—antidiabetic drug, NA—no adjustment, M850/24—metformin (850 mg/24 h), M1/12 + S50/12—metformin (1000 mg/12 h) + sitagliptin (50 mg/12 h), M850/12—metformin (850 mg/12 h), M850/24 + E10/24 + IAI—metformin (850 mg/24 h) + empagliflozin (10 mg/24 h) + intermediate-acting insulin (50 UI/day), M850/12 + S50/12—metformin (850 mg/12 h) + sitagliptin (50 mg/12 h), BMI—body mass index, RA—rheumatoid arthritis, MDA—moderate disease activity, HDA—high disease activity, DAS28-ESR—Disease Activity Score 28-joint count using Erythrocyte Sedimentation Rate, DAS28-CRP—Disease Activity Score 28-joint count using C-Reactive Protein, CDAI—Clinical Disease Activity Index, SDAI—Simplified Disease Activity Index, Rem—remission, LDA—low disease activity, GC—glucocorticoid (prednisone dose equivalent), and MTX—methotrexate.

**Table 3:** Main findings following 6 months of treatment with baricitinib.

Parameters	BARI-1	BARI-2	BARI-3	BARI-4	BARI-5	BARI-6	BARI-7	*p*-Value
Sex	F	F	M	M	F	M	F	
Age (years)	58	54	71	73	80	63	73	
HbA1c (%)								
Prior to BARI	8.2	7.6	7.4	6.5	6.4	7.6	7.9	0.035
At 6 months	6.8	6.5	6.6	6.4	6.0	7.6	6.8	
AD dosage	NA	DD	NA	NA	DD	NA	NA	
Prior to BARI	M850/12	M850/12	P15/24 + IAI	M1/24 + P30/24 + LAI	M850/12	M850/24	M850/24	
At 6 months	M850/12	M850/24	P15/24 + IAI	M1/24 + P30/24 + LAI	M850/24	M850/24	M850/24	
BMI (kg/m^2^)								
Prior to BARI	28.4	30.7	35.6	28.7	22.2	26.8	34.0	0.022
At 6 months	28.7	33.0	36.4	30.0	22.6	27.2	34.0	
RA disease activity								
Prior to BARI	MDA	HDA	MDA	MDA	MDA	MDA	MDA	-
DAS28-ESR	5.08	6.44	5.06	4.31	3.93	4.50	5.05	
DAS28-CRP	4.48	7.14	4.39	3.14	4.07	3.59	4.23	
CDAI	22.00	54.00	15.00	14.00	16.00	16.00	15.00	
SDAI	25.80	55.00	15.90	14.10	18.40	16.40	12.80	
At 6 months	Rem	LDA	Rem	LDA	LDA	LDA	LDA	
DAS28-ESR	2.28	3.17	0.28	3.05	2.96	3.10	3.06	
DAS28-CRP	2.28	2.29	1.89	2.44	2.63	2.49	2.59	
CDAI	2.00	10.00	2.00	7.00	8.00	8.00	5.00	
SDAI	2.70	10.50	2.50	7.20	8.70	8.20	6.20	
RA treatment								
GC (mg/day)								
Prior to BARI	0.0	0.0	5.0	0.0	5.0	5.0	5.0	0.317
At 6 months	0.0	0.0	5.0	0.0	2.5	5.0	5.0	
MTX (mg/week)								
Prior to BARI	5.0	0.0	0.0	0.0	0.0	5.0	0.0	-
At 6 months	5.0	0.0	0.0	0.0	0.0	5.0	0.0	

Differences were evaluated utilizing the Wilcoxon test. *p*-value < 0.05. BARI—baricitinib, F—female, M—male, HbA1c—glycosylated hemoglobin, AD—antidiabetic drug, NA—no adjustment, DD—dose decrease, M850/12—metformin (850 mg/12 h), P15/24 + IAI—pioglitazone (15 mg/24 h) + intermediate-acting insulin (60 UI/day), M1/24 + P30/24 + LAI—metformin (1000 mg/24 h) + pioglitazone (30 mg/24 h) + long-acting insulin (10 UI/day), M850/24—metformin (850 mg/24 h), BMI—body mass index, RA—rheumatoid arthritis, MDA—moderate disease activity, HDA—high disease activity, DAS28-ESR—Disease Activity Score 28-joint count using Erythrocyte Sedimentation Rate, DAS28-CRP—Disease Activity Score 28-joint count using C-Reactive Protein, CDAI—Clinical Disease Activity Index, SDAI—Simplified Disease Activity Index, Rem—remission, LDA—low disease activity, GC—glucocorticoid (prednisone dose equivalent), and MTX—methotrexate.

## Data Availability

The datasets used and/or analyzed during the current study are available from the corresponding author on reasonable request.
